# Publications From Saudi Arabia in Orthopedic Surgery in the Recent Six Years: A Systematic Review

**DOI:** 10.7759/cureus.87326

**Published:** 2025-07-05

**Authors:** Mashael M Muwanis, Danah O Sandaqji, Atheer M Aljohani, Khalid M Alkhalifah, Afnan M Akhwan, Hind N Alsubaiyi, Ghaidaa A Almuhammadi

**Affiliations:** 1 Surgical and Surgical Specialty Departments, Taibah University, Medina, SAU; 2 Faculty of Medicine, Taibah University, Medina, SAU; 3 Ear, Nose, Throat Department, Al-Rass General Hospital, Qassim Health Cluster, Al-Rass, SAU

**Keywords:** orthopaedic, orthopaedic surgeons, publications, research, saudi arabia

## Abstract

To evaluate the trends, quality, and scope of orthopedic research output from Saudi Arabia between 2017 and 2022, providing insights for future research planning and development. A systematic search was conducted on the PubMed database to identify English-language orthopedic publications published from Saudi Arabia between January 2017 and December 2022. Articles were screened in stages and categorized based on subspecialty, study design, geographical origin, and citation metrics. Data analysis was performed using Stata 15 (Stata Corp LLC, Texas, USA). A total of 348 articles met the inclusion criteria. The annual publication rate increased significantly over the six years, peaking in 2022. The most frequently studied subspecialty was spine surgery, accounting for 75 articles (21.5%), followed by arthroplasty with 69 articles (19.8%). Case reports with 98 articles (28.1%) and cross-sectional studies with 90 articles (25.8%) were the most common study designs. Geographically, the central region produced the majority of publications, with 208 articles (59.7%). Citation analysis revealed the highest impact in spine surgery (H-index = 12), followed by sports medicine (H-index = 10) and pediatric orthopedics (H-index = 9). Orthopedic research in Saudi Arabia has shown steady growth, with particular strengths in spine and sports research. However, there is a need for broader subspecialty representation, enhanced research quality, and greater geographic diversity. Strategic investments in research infrastructure, mentorship, and high-impact studies are essential to elevate the visibility and clinical relevance of Saudi orthopedic research.

## Introduction and background

Saudi Arabia has a population of approximately 32.18 million, making it the largest country in the Arabian Peninsula [1]. Notably, the country boasts a rich culture of learning and research [2,3]. Investing in medical and clinical research and development activities is imperative to generate a substantial volume of studies, facilitating sustained and economically viable growth [4].

Orthopedics is a surgical specialty that focuses on the diagnosis, treatment, and management of disorders affecting the musculoskeletal system. It is an expanding field that demands comprehensive ongoing research to enhance physicians’ knowledge and patients’ quality of life [5]. 

Medical research publications constitute an important aspect of any country’s health service improvement [6]. They are a standard way for researchers to share their findings with healthcare providers and scientists in related fields. The cross-disciplinary exchange of information leads to better management and healthcare for patients and fuels innovation in medical research [6]. Given that orthopedic conditions are noticeably common in Saudi Arabia [7,8], recognizing research gaps and areas that need improvement is a critical step for the development of future orthopedic research projects. This allows researchers to focus on areas that have not been comprehensively explored and avoid repeat studies, thereby improving the overall care, satisfaction, and quality of life of patients [9,10].

Therefore, this study was conducted to comprehensively evaluate and assess all published articles on orthopedic research from Saudi Arabia in the six-year period between 2017 and 2022. The study provides an in-depth analysis of the current research situation, including the quantity and quality of orthopedic research output in the country, and highlights research gaps that require further attention and improvement in terms of orthopedic subspecialties, geographic regions, and study designs.

## Review

Material and methods

Through the Institute for Scientific Information (ISI). Our electronic search was conducted on PubMed using the following search terms: "orthopaedic/orthopaedics AND KSA (Kingdom of Saudi Arabia)" and "orthopaedic/orthopaedics AND Saudi Arabia." We also searched by individual orthopedic subspecialties using the following search terms: ‘Saudi Arabia AND [subspecialty]’ and ‘KSA AND [subspecialty]’. Subspecialty terms included arthroplasty, foot and ankle, oncology, spine surgery, sports medicine, trauma, pediatrics, hand and upper extremity, shoulder and elbow surgery, and limb lengthening. A total of 2487 articles were identified, and after deduplication, 1478 articles remained. 

The inclusion criteria were as follows: Orthopaedic studies published in either Saudi or international journals between January 2017 and December 2022, conducted by Saudi researchers, and focused on the Saudi Arabian population. Multicenter studies that included Saudi Arabia as a participating center were also included. Only articles published in English were considered. Studies were excluded if they were not conducted by Saudi researchers, did not involve the Saudi population, were outside the specified time frame, were not published in English, or involved cadaveric subjects, non-clinical subjects, or veterinary orthopedics. 

In the first level of screening, two authors screened the titles and years of publication of all articles in a blind manner, and any conflicts were resolved by the research supervisor as a third member. 

In the second level of screening, the same two authors reviewed the abstracts of articles that passed the first level of screening. A total of 639 articles remained at this stage. 

**Figure 1:**
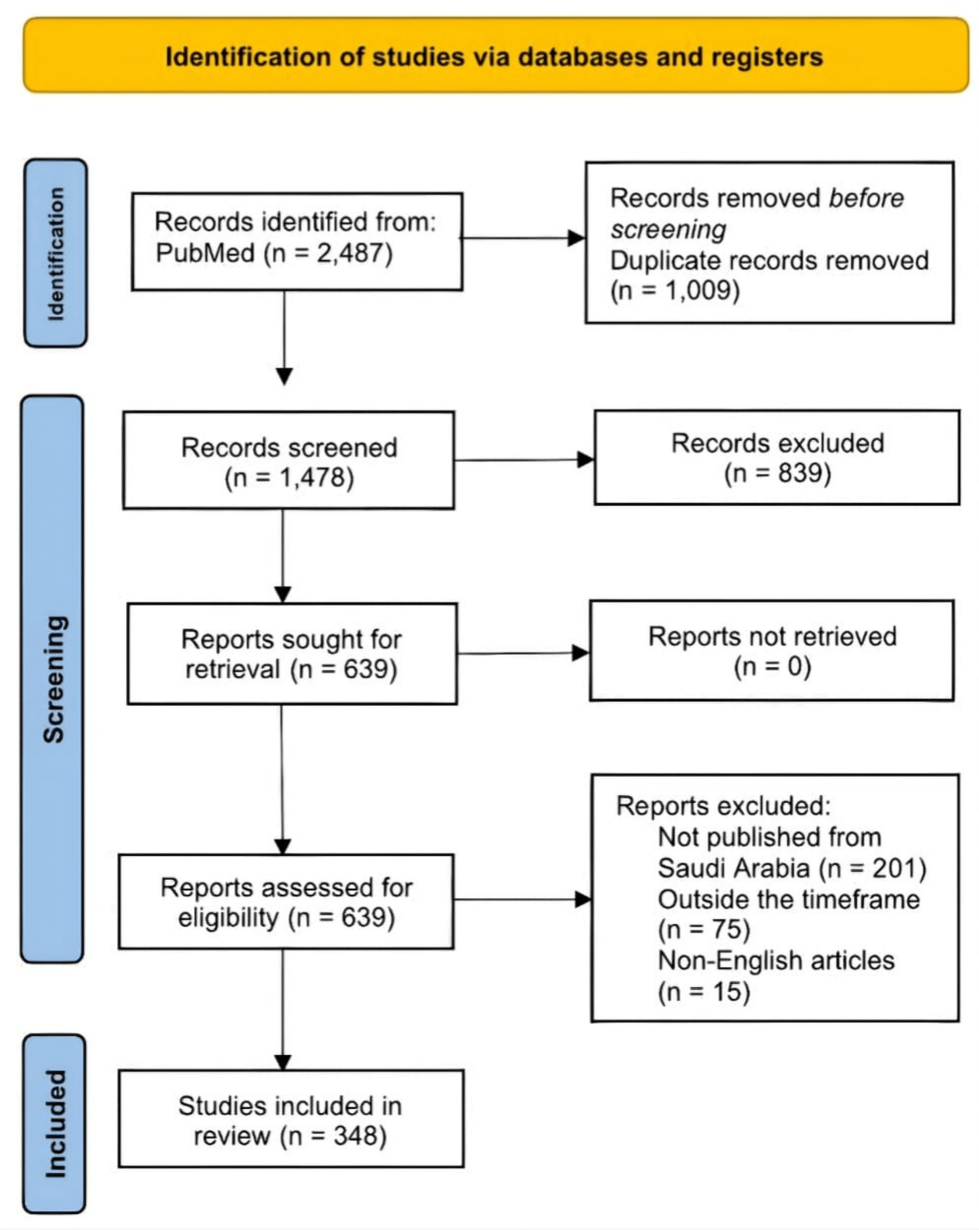
PRISMA flow chart. The image is created by the author.

In the final screening stage, blinding was removed, and the full texts were screened, and a final 348 articles were included. The details of study selection are provided in the PRISMA flow chart in Figure 1. After that, the included articles were reviewed for the purpose of classification. Each article was categorized according to geographical region (central, eastern, western, southern, and northern), orthopedic subspecialty (general orthopedics, arthroplasty, foot and ankle, oncology, spine surgery, sports, trauma, pediatrics, hand and upper extremity, shoulder and elbow, and limb lengthening), study type (case report, case-control, cross-sectional, retrospective or prospective cohort, randomized controlled trial (RCT), review article, and systematic review and meta-analysis), year of publication, and publishing journal. All collected data were analyzed using Stata 15 (Stata Corp LLC, Texas, USA). 

Results

As shown in Figure 1, during the research period, from January 2017 to December 2022, 348 articles were published, amounting to an average of 58 articles per year. 

**Figure 2:**
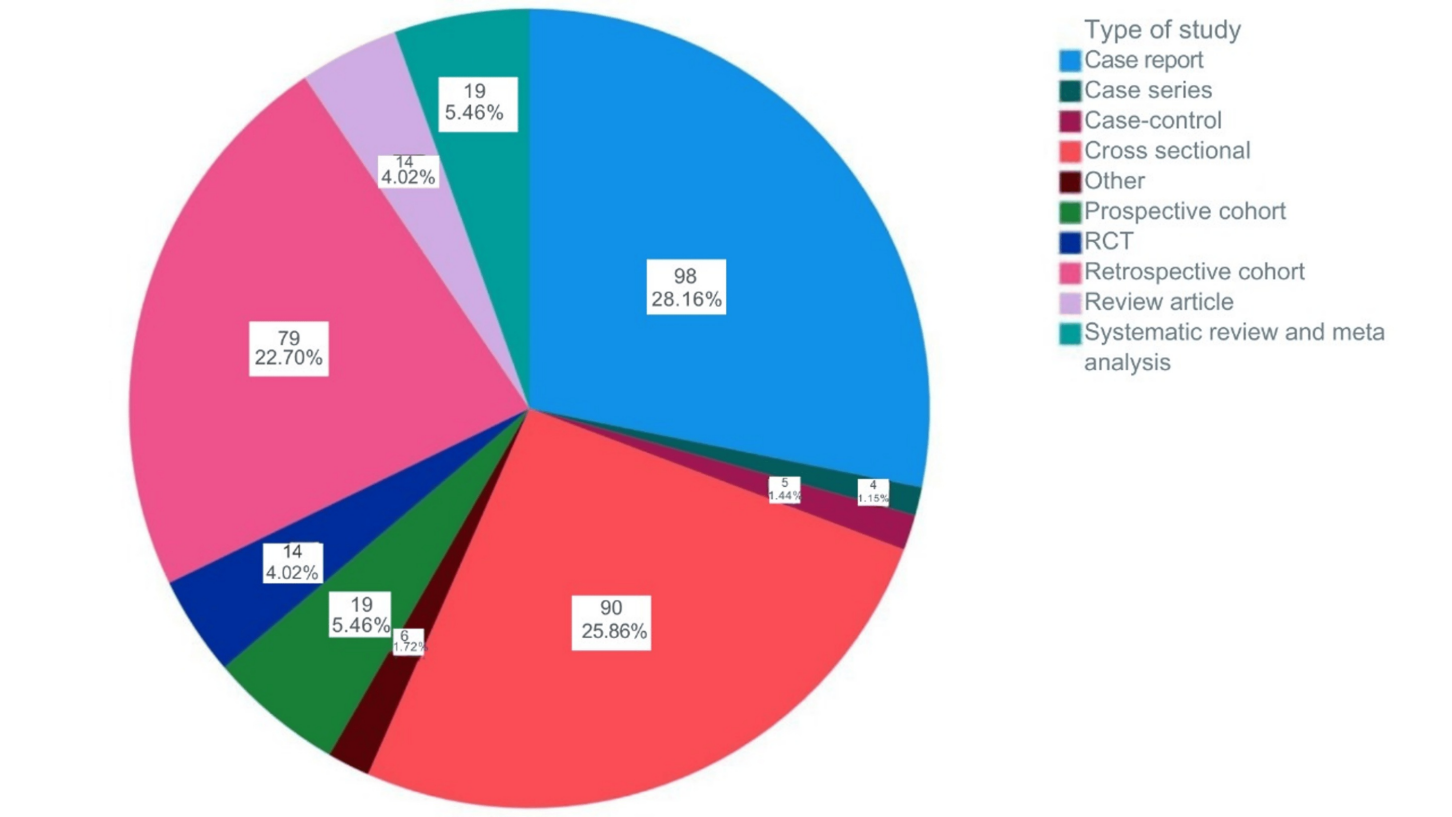
Number and percentage of publications according to study designs. The image is created by the author.

As shown in Figure 2, the most common study types were case reports with 98 articles (28.1%), followed by cross-sectional studies with 90 articles (25.8%), retrospective cohort studies with 79 articles (22.7%), systematic reviews and meta-analyses with 19 articles (5.4%), prospective cohort studies with 19 articles (5.4%), 14 review articles (4%), 14 RCTs (4%), seven retrospective case-control studies (2%), and seven case series (2%). The trend shows that case reports were the most frequent over the past six years, whereas systematic reviews/meta-analyses and prospective cohort studies were less common.

**Figure 3:**
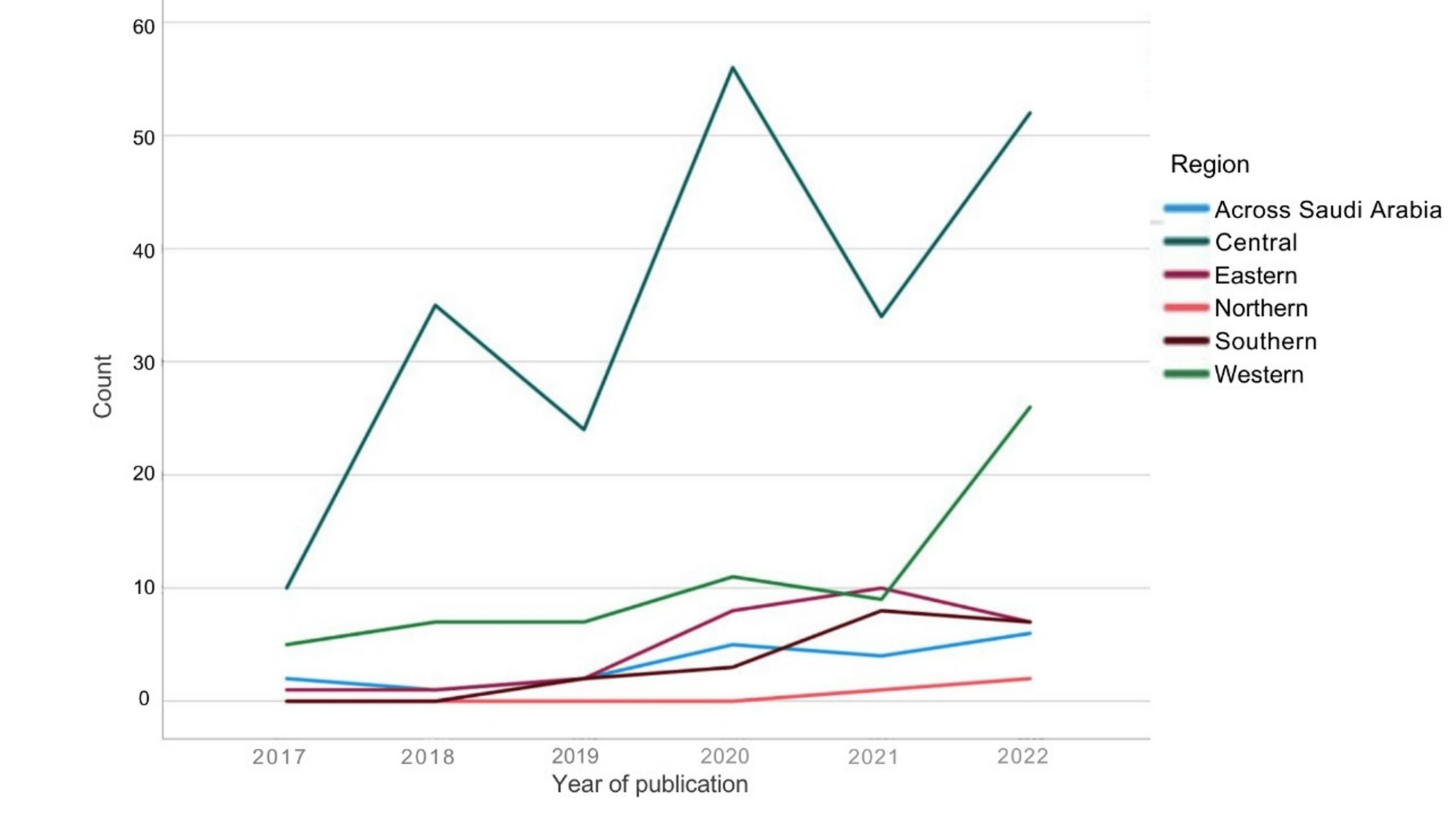
Number of publications per year from each geographical region. The image is created by the author.

**Figure 4:**
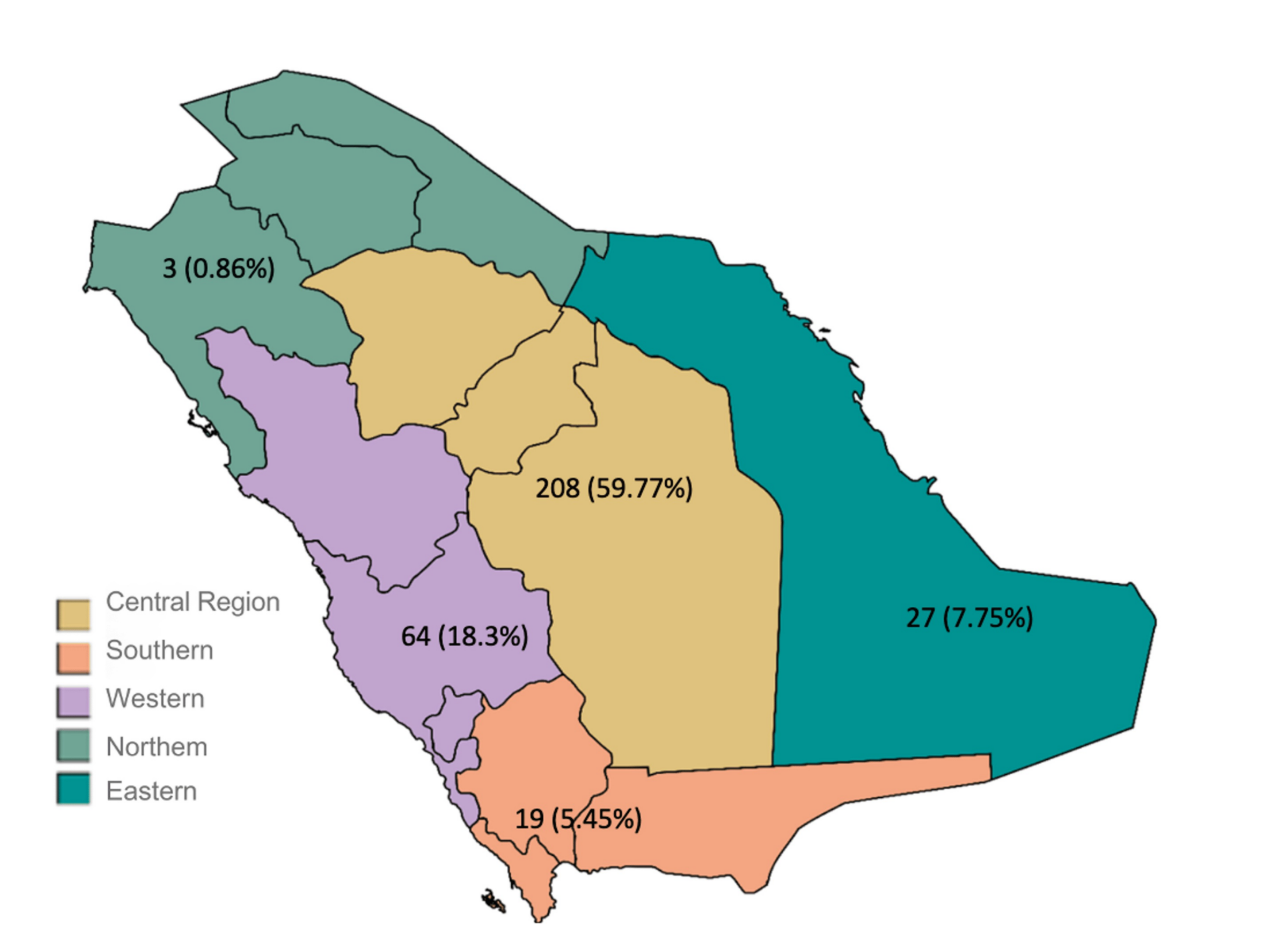
Number of publications from each geographical region. Image credits: Danah Sandaqi by Microsoft Excel. No external sources were used for this figure.

Figures 3, 4 show that in terms of geographical distribution, most articles were published by authors from the central region, 208 articles (59.7%), followed by the western region, 64 articles (18.3%), the eastern region, 27 articles (7.7%), the southern region, 19 articles (5.4%), and the northern region, three articles (0.8%). 

**Figure 5:**
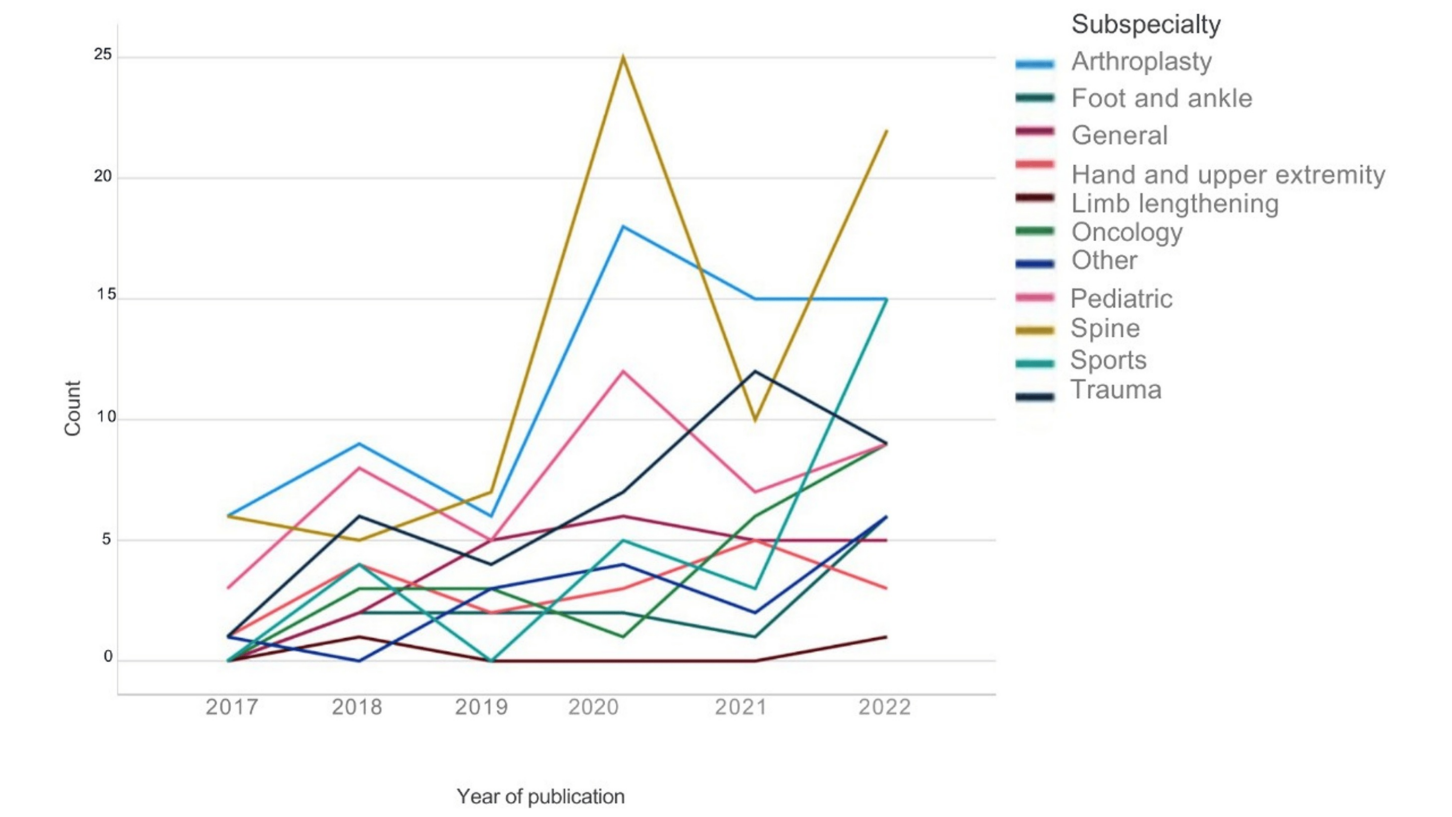
Number of publications per year according to orthopedic subspecialties. The image is created by the author.

As shown in Figure 5, the most common orthopedic subspecialty was spine surgery, with 75 articles (21.5%), followed by arthroplasty, 69 articles (19.8%); pediatric surgery, 44 articles (12.6%); trauma surgery, 39 articles (11.2%); sports surgery, 27 articles (7.7%); general orthopedic surgery, 23 articles (6.6%); oncology, 22 articles (6.3%); hand/upper extremity surgery, 18 articles (5.1%); limb lengthening, 18 articles (5.1%); and foot and ankle surgery, 13 articles (3.7%).

Table 1 shows the citation analysis of the included orthopedic articles, which displayed variability in impact across subspecialties. Articles related to spine surgery had the most citations, with an H-index of 12, suggesting that 12 articles in the list have been cited at least 12 times each. The H-indices of articles related to other subspecialties were as follows: sports medicine (H-index = 10); pediatric orthopedics (H-index = 9); oncology, foot/ankle surgery, and trauma surgery (H-index = 7-8); arthroplasty, hand/upper extremity surgery, and limb lengthening (H-index = 6); and general orthopedics (H-index = 3). The overall H-index of all articles was 22, suggesting that articles related to orthopedic research published in Saudi Arabia have been successful in disseminating knowledge at the international level.

**Table 1:** The H-index for each orthopedic subspecialty and the H-index for total publications from Saudi Arabia.

Subspecialty	H-index
Limb lengthening	6
Arthroplasty	6
Foot and ankle	7
General orthopedics	3
Hand and upper extremity	6
Oncology	7
Pediatric	9
Spine	12
Sports	10
Trauma	8
Orthopedic publication in SA	22

Discussion

Research is essential for advancing medicine and improving healthcare globally [10,11]. Like other countries, Saudi Arabia’s contribution to medical research has grown recently [3]. Although still evolving, the volume of publications related to orthopedic surgery in Saudi Arabia showed consistent growth during the studied six years, increasing from 19 articles in 2017 to 105 articles in 2022, averaging 58 articles annually. While overall publication volume increased, certain subspecialties showed different trends. Sports-related publications increased substantially in 2022 compared to previous years. However, subspecialties like trauma, spine, and pediatrics peaked in 2020 rather than 2022, highlighting variable research activity across different areas. This increase in publications in recent years suggests good development in the field. Recent studies have addressed knowledge gaps related to the epidemiology of orthopedic conditions in Saudi Arabia, evaluated the outcomes of different operative techniques and implants, and detailed innovative approaches to treat complex cases like challenging multi-stage revision arthroplasty and re-revision surgeries, prosthetic joint infections, and difficult spinal deformity corrections in adults and pediatrics. Other research assessed patient-reported outcomes after orthopedic procedures. This wealth of new research provides valuable insights into the unique orthopedic needs and challenges of the Saudi population [11]. 

In limb lengthening research, almost all 18 studies (5.1%) focused on optimizing pain control during lower limb deformity correction and enhancing bone formation through distraction osteogenesis techniques. Hand and upper extremity research included 14 case reports (4.0%), with emphasis on unusual dislocations such as carpal bone fracture-dislocations and congenital deformities, while four articles (1.1%) discussed neurovascular and brachial plexus injuries. Spine surgery literature comprised 22 cross-sectional surveys (6.3%) evaluating low back pain prevalence and associated risk factors in the Saudi population, along with retrospective cohorts analyzing postoperative outcomes and complications of certain spine diseases such as scoliosis, which was studied in 14 articles (4.0%), and disc-related conditions, which were studied in 11 articles (3.2%). In pediatric orthopedic research, 21 studies (6.0%) focused on developmental dysplasia of the hip, including risk factors and prevalence in Saudi Arabia, while fewer other studies discussed pediatric congenital anomalies such as clubfoot. Many of these articles were retrospective in design.

In the domain of foot and ankle surgery, 4 studies (1.1%) were identified that included case reports on rare massive coalitions, three systematic reviews (0.9%) on conservative management for Achilles tendon rupture, and six cross-sectional studies (1.7%) on plantar heel pain. Sports medicine research accounted for approximately 20 studies (5.7%) focusing on anterior cruciate ligament (ACL) reconstruction techniques, graft choices, rehabilitation strategies, return-to-play outcomes, and postoperative complications.

In arthroplasty, 37 studies (10.6%) focused specifically on total knee replacement (TKR), encompassing postoperative evaluations, intraoperative performance, preoperative assessments, and awareness studies. Additionally, two articles (0.6%) reported rare complications such as wrong-side implantation and prosthetic joint infections. Furthermore, six studies (1.7%) were dedicated to the development of Arabic versions of validated joint outcome instruments, such as the Harris Hip Score and other knee scoring systems, which are crucial for culturally sensitive assessment in Arabic-speaking populations.

Complex cases were featured across 12 studies (3.4%) and typically referred to multi-stage revision surgeries and periprosthetic infections and cases that required individualized surgical planning, such as total hip replacement and related complications in patients with sickle cell disease and developmental dysplasia of the hip complicated by arthritis or avascular necrosis (AVN). Orthopedic oncology research included seven studies (2.0%) that addressed diagnostic approaches, and five studies (1.4%) focused on management strategies for benign and malignant bone tumors, along with 10 case reports and case series (2.9%) that discussed rare presentations of tumors such as giant cell tumors and aneurysmal bone cysts. 

Trauma publications represented 20 studies (5.7%) investigating unique fracture patterns and their management, 11 studies (3.2%) analyzed postoperative complications and rehabilitation outcomes, and eight studies (2.3%) provided hospital cost analysis. 

Case reports represented the most common publication type, with 98 articles (28.1%), consistent with the article type published in other surgical fields in Saudi Arabia. This could be because case reports are relatively quick and straightforward to publish compared to large retrospective studies or clinical trials, which require significant time, funding, and infrastructure [12]. Most Saudi orthopedic surgeons work in a busy clinical setting, with limited time and support for research [13]. More national funding and research mentoring could facilitate advanced research engagement. Notably, this publication trend contrasts with international data. In a 10-year bibliometric analysis by Zou et al. [14], case reports formed a smaller fraction of total orthopedic research output in leading countries such as the USA, UK, Germany, Japan, and Mainland China. The USA dominated the overall research volume (24.2% of global orthopedic output from 2005 to 2014) and led in nearly all study types, including clinical trials and reviews, while case reports were proportionally less prominent. In Mainland China, although total publication volume grew rapidly, the focus was increasingly on meta-analyses and randomized controlled trials rather than case reports, reflecting a national effort to improve research quality. These international trends highlight the gap in research infrastructure and support between Saudi Arabia and more research-intensive countries. 

In the central region, which produced 208 (59.7%) of publications, likely reflecting the abundance of universities, hospitals, training programs, and clinical workload in this region, there are eight major academic medical centers with established orthopedic departments, yielding a publication ratio of 26 articles per center [15]. The western region, with four academic centers, produced 64 (18.3%) of publications, averaging 16 publications per center. The eastern region, with two centers, contributed to 27 (7.7%) publications, averaging 13 publications per center. The southern region's two centers produced 19 (5.4%) publications, averaging nine publications per center, while the northern region's single center produced three (0.8%) publications. This analysis reveals that beyond raw publication numbers, there are significant differences in research productivity per academic center across regions [13,11].

Spine surgery was the most published topic, with 75 articles (21.5%), likely due to its overlap with other specialties like neurosurgery and specific research interests. This topic was followed by arthroplasty with 69 articles (19.8%) and pediatrics with 44 articles (12.6%). Few articles were published related to hand and upper extremity surgery, oncology, and foot and ankle surgery.

Although all articles had a moderate collective citation index, citation analyses revealed that certain fields, such as spinal surgery, pediatric surgery, and sports medicine, have particularly good international acceptance. Further promotion of excellence in these emerging fields and popularizing good-quality research across all domains could boost Saudi Arabia's global thought leadership and benefit patient communities worldwide. While the number of articles published has increased, there is room for improvement in terms of research quality, geographic representation, subspecialty breadth, and international impact [16]. Targeted efforts to enrich research methodology, engage young researchers, incentivize collaborative research, and promote high-quality studies across all orthopedic focus areas could substantially improve publication metrics in the field of orthopedics [17-19].

To assess the global position and research potential of Saudi Arabia, we compared our findings with similar articles from other countries. These included articles from the Middle East, China, the USA, the UK, Japan, and Germany [20,14]. In terms of articles from the Middle East published between 2013 and 2017, there were 481 articles from Egypt with a total of 1243 citations. Most published articles were on trauma (22%), followed by arthroscopy (16%) and hand and microsurgery (15%). Only 3% of the articles were published on deformity. There was a gradual increase in the number of articles published each year except for the topic of spine surgery, for which the number remained constant [20]. 

Another review conducted over a period of 10 years (2005-2014) on orthopedic articles from the USA, UK, Japan, Germany, and China revealed a total of 128,895 articles [14]. The USA contributed the highest number of studies (24.2%), whereas China had the lowest number of articles published (2.6%). However, there was a decrease in the number of articles from the USA over time, whereas the number kept increasing for other countries.

In terms of study types, the USA had the greatest number of all types of articles except for meta-analyses, which were mostly published by authors from China. Review studies were the most common type of study, whereas meta-analyses were the least common.

Considering the total number of citations, the order from highest to lowest was as follows: USA, UK, Germany, Japan, and China. Articles from the USA had 481,363 citations, whereas articles from China had only 25,787. In terms of impact factor (IF), again, articles from the USA ranked highest with an average IF of 2.191, whereas articles from the UK ranked the lowest with an average IF of 1.802. The remaining countries are ranked in the following order: Germany, Japan, and China [14].

In general, when comparing Saudi Arabia to other regions worldwide, the total number of articles published annually was relatively good compared to the number of articles published annually from other Middle Eastern countries [14], but lower than that on a global scale. In contrast to other Middle Eastern countries, trauma was less commonly studied in Saudi Arabia, along with hand and upper extremity surgery. This highlights a need for increased research on these topics. Arthroplasty and spine surgery were comprehensively studied, but the need for further improvement remains in terms of quality, as the number of citations was low.

An analysis of the impact factor (IF) of the journals in which the included articles were published revealed that the highest IF was 4.6, with a total of 31 articles (9%) published in journals with an IF that equals or is greater than 4. Also, 62 articles (17.8%) were published in journals with an IF between 3 and 3.9. The majority, representing 237 articles (68%), were published in journals with an IF between 1 and 2.9. Only a small proportion, 18 articles (5%), were published in journals with an IF less than 1 [20]. When comparing our results to countries such as the USA, UK, Japan, and Germany, which had average IFs of journals as 2.19, 1.8, 2.1, and 2.2, respectively [14], Saudi Arabia could be considered equal in terms of the quality of the journals in which the articles were published. This is a positive outcome and indicates that Saudi Arabia is at par with countries worldwide. 

## Conclusions

This systematic review demonstrates a significant upward trend in orthopedic research output from Saudi Arabia over the past six years, reflecting the country’s growing momentum and potential to lead regionally in this field. Most publications were concentrated on spine surgery and arthroplasty, with a predominance of case reports and cross-sectional studies. While other specialties like foot and ankle, orthopedic oncology, and study designs such as clinical trials, cohort studies, and systematic reviews were the least popular in research output. This uneven distribution across subspecialties and regions reveals disparities in research engagement. These findings underscore the need for a strategic shift toward studies with high methodological standards and quality, balanced subspecialty representation, and broader national participation. Strengthening research infrastructure, fostering mentorship, and encouraging collaborative, multi-center studies will be essential to enhance the quality, impact, and global visibility of orthopedic research in Saudi Arabia.
